# The Lonely Brain – Associations Between Social Isolation and (Cerebro-) Vascular Disease From the Perspective of Social Neuroscience

**DOI:** 10.3389/fnint.2022.729621

**Published:** 2022-01-28

**Authors:** Janine Gronewold, Miriam Engels

**Affiliations:** ^1^Department of Neurology, Center for Translational Neuro- and Behavioral Sciences (C-TNBS), University Hospital Essen, University of Duisburg-Essen, Essen, Germany; ^2^Institute of Medical Sociology, Medical Faculty, Heinrich-Heine-University Düsseldorf, Düsseldorf, Germany

**Keywords:** COVID-19, social isolation, stroke, cardiovascular diseases, interoception, psychological adaptation, physiological adaptation, neurological models

## Abstract

The current COVID-19 pandemic led to a considerable reduction in in-person social contacts all over the world. In most individuals, reduced social contacts lead to the perception of social isolation causing feelings of loneliness, which are experienced as stressful. Experiencing social distress due to actual or perceived social isolation has been associated with negative health outcomes such as depression, (cerebro-) vascular disease and mortality. Concrete mechanisms behind this association are still a matter of debate. A group of researchers around Hugo Critchley with special contributions of Sarah Garfinkel and Lisa Quadt proposes a framework for the underlying brain-body interactions including elements from models of social homeostasis and interoceptive predictive processing that provides important insights and testable pathways. While in a previous publication, we reviewed literature on the observed association between social isolation and stroke and coronary heart disease, we now extent this review by presenting a comprehensive model to explain underlying pathomechanisms from the perspective of social neuroscience. Further, we discuss how neurodivergent people, e.g. autistic individuals or persons with attention deficit disorders, might differ in these pathomechanisms and why they are especially vulnerable to social isolation. Finally, we discuss clinical implications for the prevention and therapy of (cerebro-) vascular diseases.

## Introduction

The current COVID-19 pandemic leads to considerable changes in professional and personal life in people from all over the world. Lockdowns and physical distancing require many people to work from home or work short hours, some even lose their jobs. Reductions in in-person social contacts due to changes in professional life are accompanied by social changes in personal life: in-person social contacts including meeting family members and friends or engaging in group activities such as sports clubs are restricted to limit spreading of the SARS-CoV-2 virus. Consequently, the COVID-19 pandemic transforms social interactions. While in-person contacts decrease, virtual contacts via video calls, audio calls or text messages increase. Compared to in-person contacts, virtual contacts provide fewer non-verbal cues, greater potential for anonymity, more opportunity to form new social ties and bolster weak ties, and wider dissemination of information (Lieberman and Schroeder, 2020). Especially the restricted potential to deliver and receive non-verbal information such as touch, smell, the exact modulation of one’s voice, or movement patterns impairs social interaction, provokes miscommunication and reduces the feeling of connection (Kruger et al., 2005; Hall and Schmid Mast, 2007). Consequently, the restriction of in-person contacts during the COVID-19 pandemic decreases the quality of social interactions which can increase the feeling of loneliness. First analyses from longitudinal population-based studies support this hypothesis. In the German National Cohort, an Austrian older sample, and a biracial American sample for example, an increase in loneliness was observed following the pandemic onset (Berger et al., 2021; Kucharska-Newton et al., 2021; Mayerl et al., 2021), which was related to the perceived COVID-19 related social restrictions (Mayerl et al., 2021). Having a low number of social contacts (social isolation) as well as perceiving a lack of social contacts or a low quality of social contacts (loneliness and lack of social support) has negative influences on mental and physical wellbeing (Quadt et al., 2018, 2020; Berger et al., 2021; Kucharska-Newton et al., 2021; Mayerl et al., 2021) including depression (Erzen and Çikrikci, 2018; Berger et al., 2021; Mayerl et al., 2021; Van As et al., 2021), stroke and myocardial infarction (Gronewold et al., 2020, 2021; Gronewold and Hermann, 2021), and mortality (Vogt et al., 1992; Stringhini et al., 2012; Steptoe et al., 2013; Rico-Uribe et al., 2018; Gronewold et al., 2020; Ward et al., 2021). Performing a comprehensive review and meta-analysis, Valtorta et al. (2016) observed that poor social relationships (including social isolation and loneliness) were associated with a 29% increase in risk of incident CHD and a 32% increase in risk of incident stroke. Three main mechanisms underlying these observations were discussed, that is behavioral (such as physical inactivity, smoking, and alcohol abuse), psychological (such as low self-esteem, limited coping mechanisms, and negative affectivity) and physiological mechanisms (such as disturbed immune functioning and dysregulation of vascular risk factors like blood pressure) (Valtorta et al., 2016). Recently, an innovative framework to decipher pathomechanisms underlying the negative health outcomes of social isolation and loneliness was developed by Quadt et al. (2020) combining the social allostasis model (Matthews and Tye, 2019) with ideas of interoceptive predictive processing (Barrett and Simmons, 2015). Based on our previous studies analyzing the association of social isolation with stroke and coronary heart disease (Gronewold et al., 2020, 2021; Gronewold and Hermann, 2021), we put a specific focus on the outcome of (cerebro-) vascular disease. We also want to highlight the increased vulnerability of neurodivergent individuals.

## Influence of Social Isolation and Loneliness on (Cerebro-) Vascular Disease

According to the framework by Quadt et al. (2020), social isolation increases the risk of (cerebro-) vascular disease by the over-activation of initially adaptive mechanisms. Social contacts represent a basic human need, which developed during evolution because living in a group protected individuals from threats of the environment and improved the chances to get access to water, food and sexual partners. From the evolutionary perspective, the feeling of loneliness evolved as a negative affect, now also called social stress or social distress, in response to isolation to draw an individual back to its group. Thus, the negative affect associated with loneliness is triggered by an adaptive response to perceived social deficits (Cacioppo et al., 2006). Only on rare occasions, social isolation is adaptive. In case of illness, isolating oneself from additional potential sources of infection as part of the sickness behavior is adaptive to foster recovery, and the feeling of loneliness represents the signal to return back to the group after recovery (Dantzer et al., 2008). Consequently, a flexible and context-dependent balance between isolation and connection is needed to ensure survival with usually seeking connection representing the most adaptive response. This balance is also termed social homeostasis.

According to predictive processing models (Barrett and Simmons, 2015), our brain coordinates this balance by comparing incoming sensory signals with predictive models about the likelihood of incoming signals in an efficient way. Before the COVID-19 pandemic, it was highly probably to see and hear large groups of people celebrating on a Friday evening in the downtown area. Going to the downtown area on the first day of a lockdown and only seeing very few people would surprise us because we are used to see large groups of people there and thus expect to see large groups based on our prediction model. In case of such a mismatch between prediction and reality, error signals occur. These error signals help the organism to detect that a specific set point associated with social needs is not met (Matthews and Tye, 2019). Error signals can either update prediction models (we do not expect to see large groups anymore, perceptual interference) or change behavior to change incoming signals (e.g. celebrate with a group of people who are SARS-CoV-2 negative, active inference) (Friston, 2012). Interoceptive predictive processing models put a focus on the sensing of internal bodily signals (Barrett and Simmons, 2015; Quadt et al., 2018). When we go to the downtown area on a Friday evening, we expect to feel a rise in blood pressure, heart rate and oxytocin release as signs of joy. Sensing these bodily sensations feels normal to us because they are within the expected range of our bodily signals. In the present times of lockdowns, where we do not meet a lot of people, we do not feel signs of joy when going to the empty downtown area on a Friday evening, but rather sense headache and tiredness.

Responding to mismatches between predicted and incoming signals, in our case between wanted and perceived social contacts, means effort for the organism. There are different homeostatic responses to perceived social deficits (Matthews and Tye, 2019). An important response is hypervigilance. Short-term increases in vigilance, arousal, and attention represent an adaptive response because it helps the socially isolated and thus more vulnerable individual to recognize and respond to potential threats from the environment. Attention is further directed toward socially relevant stimuli and increasing activity of reward processing systems like the dopaminergic system and the oxytocin system elicits motivation to reconnect. This adaptive short-term response is also known as acute stress response. The acute stress response improves information transmission, provides energy for fight-flight responses, and includes increased pro-inflammatory activity as a preparation for potential physical injury and as a consequence of the decreased risk of contagious viral infections when isolated. If social isolation cannot be resolved actively (e.g. by calling a friend), passive coping mechanisms such as attenuated emotional sensitivity ensure self-protection from emotional distress associated with isolation.

Negative health consequences of social isolation occur if the initially adaptive short-term stress response is prolonged, which occurs when the mismatch between wanted and perceived social contacts cannot be solved. Thus, error signals prevail, the individual continues to feel lonely, causing negative affect and emotional distress, and remains in a hypervigilant state. This results in allostatic overload in the long run which is not adaptive anymore but exhausts and harms the organism (McEwen, 2005). Chronic activation of the endocrine, neural and immunological mediators of the initially adaptive allostatic response aiming to achieve social homeostasis lead to systemic dysregulation of the cardiovascular, metabolic, and immune system. As a response to the dysregulation, subclinical functional deficits occur, finally resulting in clinical system damage manifested as (cerebro-) vascular disease (Peters et al., 2017). The initially adaptive short-term release of the stress hormones adrenalin and cortisol, which improves information processing, can lead to neurodegenerative processes and decreased information processing and memory function when prolonged. Glucocorticoid release secures energy supply in acute energy-demanding situations but leads to insulin resistance and increased risk for cardiovascular disease in cases of chronically increased release. Increases in blood pressure, which represent an adaptive response in situations with an acute need for increased blood flow, stress the vessel walls when repeated over a long time promoting atherosclerosis and damage of the vessel walls, particularly when combined with metabolic factors (McEwen, 2005). Natural immunity as a fast immune response, which includes release of neutrophils, macrophages, proinflammatory cytokines and natural killer cells, is increased during acute stress. This is adaptive because if the individual is harmed during fight-flight behavior, these cells can migrate to the site of injury and fight pathogens to accelerate wound repair and prevent infections. Specific immunity as a less fast but more specific response targeted to concrete stressors is decreased during acute stress to conserve energy. Chronic social stress can both increase risk for diseases associated with decreased immunity, such as infectious and neoplastic disease, and diseases associated with increased immunity, such as allergic and autoimmune disease (Segerstrom and Miller, 2004). Inflammation and infection promotes atherosclerosis, exerts prothrombotic effects, and can thus increase the risk of (cerebro-) vascular disease (Meschia et al., 2014).

## Discussion

Multiple factors can influence the vulnerability for social isolation and its (cerebro-) vascular consequences. In our previous review, we discussed the factors age, sex/gender, race/ethnicity, sexual orientation, and depression (Gronewold et al., 2021). Quadt et al. (2020) also suggest an important influence of neurodiversity. Neurodiversity represents an approach to see neurological differences as a result of normal variation in the human genome and as a social category such as gender instead of a pathology and medical disorder that needs to be cured (Armstrong, 2011; Pripas-Kapit, 2020). The neurodiversity approach was first put forward by autistic individuals and subsequently applied to a variety of other neurodevelopmental conditions (e.g. attention deficit disorders) (Jaarsma and Welin, 2012). Both in neurotypical and autistic individuals, loneliness is related to social skills and perceived quality of social contacts (Mazurek, 2014; Ee et al., 2019) and associated with depression (Mazurek, 2014).

According to previous studies, neurodivergent individuals experience loneliness and negative social contact more often than neurotypicals (Ee et al., 2019), despite longing for social contact (Müller et al., 2008). They report many barriers to socializing and that socializing with neurotypicals can be exhausting, challenging, or anxiety provoking (Müller et al., 2008; Ee et al., 2019). Differences in communication style, non-verbal social interactive cue processing and emotional expression, which lead to mutual misunderstanding between autistic and neurotypical individuals (Milton, 2012) foster social distress especially for autistic individuals, which increases their risk of (cerebro-) vascular disease. Yet, models for brain-body interactions underlying the influence of social isolation on (cerebro-) vascular health are based on research on neurotypicals and it remains to be evaluated whether the same mechanisms are involved in neurodivergent individuals (Quadt et al., 2020). Also, the above-mentioned examples are based on neurotypicals. Changes due to lockdowns might have a different influence in different individuals – while some will indeed be distressed by the lack of people in public areas, others will rather enjoy it. Emotional processing, including the sensing of own and other people’s emotion, is influenced by interoceptive processes both in autistic and neurotypical individuals (Mulcahy et al., 2019). First studies on interoception in autistic individuals show that they have lower interoceptive accuracy (Garfinkel et al., 2016), which increases the likelihood of prediction errors and allostatic overload, and can finally increase the risk for various stress-related diseases. Concordant with this theory, a large case-control study including 1507 autistic and 15070 neurotypical individuals showed that history of stroke was twice as prevalent in autistic than neurotypical individuals (Croen et al., 2015). Further research is needed to unravel reasons for increased loneliness in neurodiversity and its association with adverse health conditions such as (cerebro-) vascular disease.

## Clinical Implications

Perceived social isolation as an important psychosocial stressor, which is increasing due to reduced social contacts during prolonged lockdowns in the COVID-19 pandemic, can represent the starting point of a vicious cycle: if in-person social contacts are not controllable by the individual anymore, social needs and expectations cannot be fulfilled, which leads to a prolonged stress response including negative affect, social withdrawal and negative evaluation of social contacts as sickness behavior to conserve energy and maintain physical health. As a consequence, the brain gets into a “locked-in” state (Barrett et al., 2016), where negative prediction models about social interactions, including also predictions about internal bodily responses toward social stimuli, cannot be corrected due to reduced exposure to potentially corrective stimuli and insensitivity to prediction errors containing corrective information. This in turn causes the social environment to withdraw from the isolated individual which further increases the stress response and progression toward disease states. Thus, disease states are both a cause and consequence of social isolation (Quadt et al., 2020), which puts the prevention and treatment of social isolation and its negative consequences for mental and physical health into focus.

Even though numerous healthcare authorities recommend assessing, documenting and addressing social factors such as social isolation (Gold et al., 2019), evidence demonstrating a concrete improvement in individual and population health and a reduction in health-related costs is still scarce and completely lacking for social isolation (Gottlieb et al., 2017). Support for the validity of the social allostasis and interoception models and its educable therapy suggestions is so far provided for the outcome of depression (Mazurek, 2014). It remains to be established, whether increasing social contacts via interventions in case loneliness is detected in patients during clinical routine and at the population level, decreases the risk of (cerebro-) vascular disease. Additionally, intervention studies which focus on the promotion and quality improvement of social contacts, especially for neurodivergent individuals, are needed.

## Data Availability Statement

The original contributions presented in the study are included in the article/supplementary material, further inquiries can be directed to the corresponding authors.

## Author Contributions

JG drafted the manuscript. ME critically revised the manuscript for important intellectual content and took the lead in the associated interview with Sarah Garfinkel and Lisa Quadt. Both authors contributed to the article and approved the submitted version.

## Conflict of Interest

The authors declare that the research was conducted in the absence of any commercial or financial relationships that could be construed as a potential conflict of interest.

## Publisher’s Note

All claims expressed in this article are solely those of the authors and do not necessarily represent those of their affiliated organizations, or those of the publisher, the editors and the reviewers. Any product that may be evaluated in this article, or claim that may be made by its manufacturer, is not guaranteed or endorsed by the publisher.
